# Normal measurements of the optic nerve, optic nerve sheath and optic chiasm in the adult population

**DOI:** 10.4102/sajr.v23i1.1772

**Published:** 2019-11-05

**Authors:** Sanele S. Mncube, Matthew D. Goodier

**Affiliations:** 1 Greys Hospital, Radiology Department, College of Health Sciences, Nelson R. Mandela School of Medicine, University of KwaZulu-Natal, Pietermaritzburg, South Africa

**Keywords:** Optic nerve, optic sheath, optic chiasm, magnetic resonance imaging, MRI, anterior visual pathway

## Abstract

**Background:**

Imaging assessment of the anterior visual pathway structures, particularly the optic nerves (ON), requires knowledge of normal dimensions. Several studies suggesting techniques and normal ranges have been performed, but most suffer from various methodological flaws. This study is the first to be performed in a South African population.

**Objectives:**

The aim of this study was to establish normal measurements of the ON, optic nerve sheath (ONS) and optic chiasm (OC) on magnetic resonance imaging (MRI).

**Method:**

Eighty normal adults between ages of 12 and 65 years were included in this prospective, quantitative, observational, descriptive study to establish normal measurement of the ON, ONS and OC using a T2W 3D MRI sequence. Measurements (width and height) were undertaken by two observers independently.

**Results:**

A total of 80 participants with a mean age of 35 years were studied: 49 females (61.25%) and 31 males (38.75%). There were no statistical differences in the measurements between gender and age correlation. Interobserver agreement was best for larger structures, that is, OC width and intracranial ON width, respectively. The overall mean of OC width was 13.63 mm (range: 11.13 mm–16.92 mm, standard deviation [s.d.] 1.21); intraorbital ON height at 5 mm behind the globe 2.29 mm (range: 1.63 mm–3.33 mm, s.d. 0.43), and intracranial ON width 4.27 mm (range: 2.46 mm–5.19 mm, s.d. 0.53).

**Conclusion:**

Normal measurements of the anterior visual pathway structures on MRI are best reflected in the larger structures. Interobserver variability was poor for the orbital ON, ONS, intracranial ON height and OC height. We recommend that measurements be obtained for the OC width and intracranial ON width. The overall mean for the OC width is 13.63 mm and intracranial ON width 4.27 mm.

## Introduction

Accurate imaging assessment of the anterior visual pathway structures of a patient with abnormal vision requires knowledge of the normal dimensions of these structures. The anterior visual pathway consists of the retina, optic nerve sheath complex (ONSC), optic chiasm (OC), and optic tract. For the purposes of this article, anterior visual pathway refers to only the ONSC and OC. The ONSC comprises the optic nerve (ON) and optic nerve sheath (ONS). Disease detection affecting the anterior visual pathways may be made subjectively. However, objective measurement of these structures is often a very valuable part of the diagnostic assessment.

A spectrum of diseases may affect the size of anterior visual pathway structures.^1,2^ These comprise congenital and acquired conditions. Examples include congenital atrophic hypoplasia, optic atrophy, optic neuritis, perineuritis, glaucoma, ON glioma, optic sheath meningioma and intracranial pathology causing raised intracranial pressure (ICP) such as cryptococcal meningitis, trauma and neoplasm. Patients with abnormal vision and optic disc pallor on fundoscopic examination are often referred for imaging. Common clinical requests include evaluation of the ONs for conditions like atrophy or neuritis and intracranial pathology causing mass effect on the visual pathway structures. The World Health Organization (WHO) estimates that approximately 1.3 billion people live with some form of vision impairment globally, 80% of which is considered avoidable.^3^ The rationale for investigating patients with visual symptoms is to achieve an early diagnosis, to allow prompt intervention to maintain or restore vision and prevent permanent blindness.

Various modalities have been utilised in the past to image the ON with varying degrees of accuracy. Most of those modalities are outdated. To date, published reference values for normal ON measurements in prescribed textbooks and journal-based resources have several drawbacks. The older studies were based on computed tomography (CT) scan and low-resolution magnetic resonance imaging (MRI) images, which could not distinguish the different structures of the ONSC. The measurements therefore represent the entire ONSC rather than the actual nerve or sheath.^4,5^ Other studies do not clearly define the exact points of measurement.^4,6^

Several international publications report on various studies employing MRI for measurements of the anterior visual pathway structures. Studies by Parravano et al. and Daniels et al. looked at both the ON and OC.^4,5^ Parravano et al. concluded that measurements of the OC were more reliable than the ON. However, this study was based on low-resolution T1W MRI sequence. The remainder of the studies measured either the ON, ONS or OC.^6,7,8,9,10,11,12,13,14,15,16^

The anterior visual pathway comprises small structures. Obtaining measurements of these structures is often challenging because of inherent technical difficulty in measuring small structures. Therefore, state-of-the art imaging is necessary. High-resolution MRI based on isometric or 3D acquisition techniques is currently the best imaging modality for many applications in neuroradiological imaging and generally forms part of standard neuroradiological imaging protocols.^16,17,18^ The spatial and contrast resolution achieved by this technique typically allows for accurate evaluation of the various anterior visual pathway structures.

To date, there have been no published studies of normal MRI measurements of anterior visual pathway structures taken on a South African population. The aim of this study was to ascertain normal measurements of the anterior visual pathway structures in the normal adult population in South Africa.

## Research methods and design

This was a prospective, quantitative, observational, descriptive study conducted in the Department of Radiology at Greys Hospital, a tertiary referral institution in Pietermaritzburg, Kwa-Zulu Natal, South Africa, which serves a population of 4.5 million.

The study population comprised adult patients between the ages of 12 and 65 years, referred for MRI of the head and/or neck region without any visual signs and symptoms or central nervous system (CNS) pathology potentially affecting the visual pathway. Participants were recruited consecutively amongst adult patients referred for MRI of the head and/or neck region. An intended sample size of 73 was required to estimate ON measurements to within 0.03 mm with 95% probability, assuming a mean of 2.94 mm and standard deviation (s.d.) 0.09. A total of 100 patients were enrolled. The final sample size was 80. Twenty patients had technically inadequate examinations because of motion artefact and were excluded from the study. Other reasons for exclusion amongst the 20 were a history of pituitary surgery discovered in retrospect, suspected idiopathic intracranial hypertension and inadequate coverage of the OC.

Images were acquired with a 1.5T Ingenia Phillips MR system with the use of a multi-array head coil. Patients were positioned supine in the magnet. Straight gaze was maintained using a bright focal spot, and participants were counselled prior to the examination to avoid eye movements during image acquisition. T2W 3D TSE imaging sequence was employed. Images were processed on the workstation and archived to the Picture Archiving and Communication System (PACS). Five different MRI trained radiographers, all of whom received specific training on scanning technique for this study, performed the scans.

Two observers carried out the measurements independently. The first observer was a senior radiology trainee and the second observer was a consultant radiologist with certification in neuroradiology. The second observer was blinded to the first observer’s measurements, but not to the patients’ demographics and the inclusion criteria.

The images were viewed and analysed on PACS using Beacon G22S+ with 2 megapixel (1600 × 1200) resolution monitors. Multiplanar reconstruction (MPR) and 3D viewing functions were utilised. A digital line measurement ruler was used to perform measurements. Further electronic functions like zoom, pan and windowing were also utilised as necessary to optimise image quality and accuracy of measurements.

**FIGURE 1:**
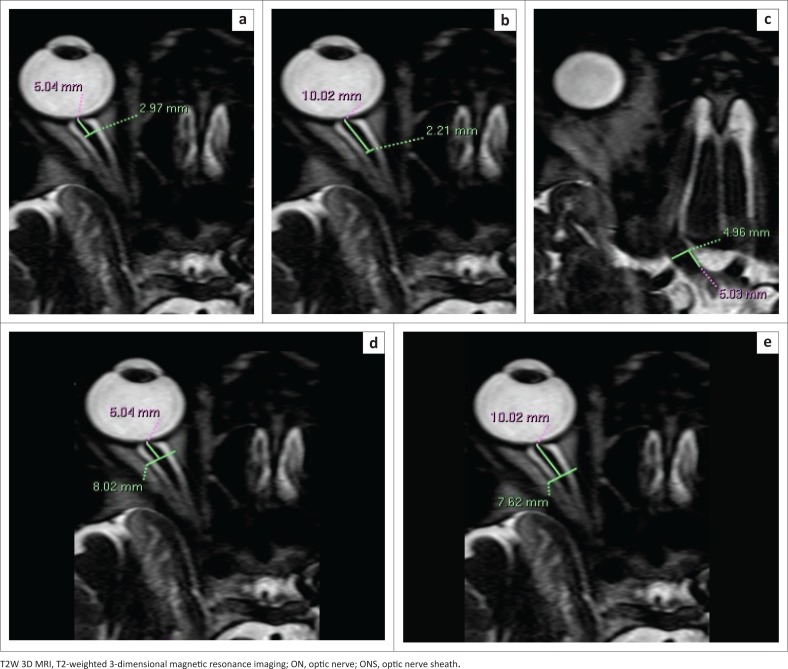
Axial T2W 3D magnetic resonance imaging of the right orbit demonstrating the measurement technique: (a and b) intraorbital optic nerve width 5 mm and 10 mm behind the globe, respectively; (c) intracranial optic nerve width and (d and e) optic nerve sheath width 5 mm and 10 mm behind the globe, respectively.

**FIGURE 2:**
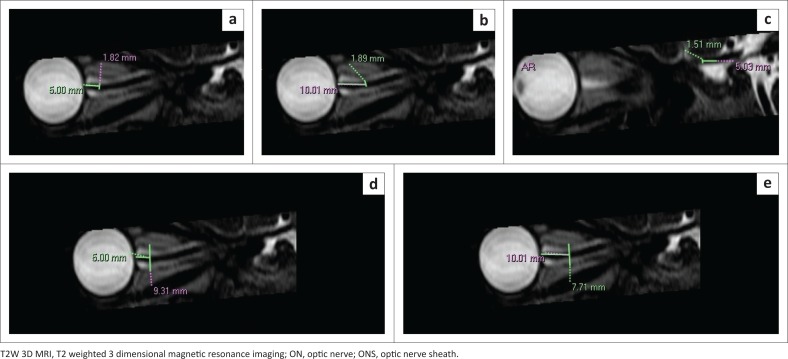
Reformatted sagittal T2W 3D magnetic resonance imaging of the right orbit demonstrating the measurement technique: (a and b) intraorbital optic nerve height 5 mm and 10 mm behind the globe, respectively; (c) intracranial optic nerve height and (d and e) optic nerve sheath height 5 mm and 10 mm behind the globe, respectively.

**FIGURE 3:**
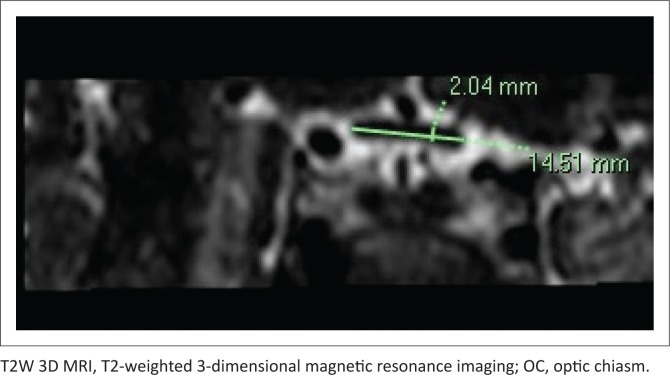
Reformatted coronal T2W 3D magnetic resonance imaging demonstrating the measurement technique of the optic chiasm width and height.

Measurements were conducted on the right side only. Width and height of the ON and ONS were obtained on axial and reformatted sagittal images, respectively, and width and height of the OC were obtained on reformatted coronal images. The ON was measured at three points: intraorbital segment (5 mm and 10 mm behind the globe) and the intracranial segment (5 mm from the chiasm) (Figures 1 and 2). Optic nerve sheath measurements were performed at two segments: 5 mm and 10 mm behind the globe (similar points as for the intraorbital ON). Finally, measurements of the OC were obtained at a single point on reformatted coronal images (Figure 3).

Data were collected and recorded in customised Microsoft Excel tables. Analysis was performed using the Stata V13 program. Descriptive statistics were used to describe the demographic characteristics of the sample. Frequency distributions and percentages were used to summarise categorical data such as age, sex, width, and length. Ranges, means (in millimetres) and standard deviations were calculated and used to summarise the data. Interobserver reliability was tested with the Bland and Altman method, and presented graphically.

### Ethical consideration

Full ethics approval was obtained from the University of KwaZulu-Natal Biomedical Research Ethics Committee (BREC). Reference number: BE442/18.

## Results

**FIGURE 4:**
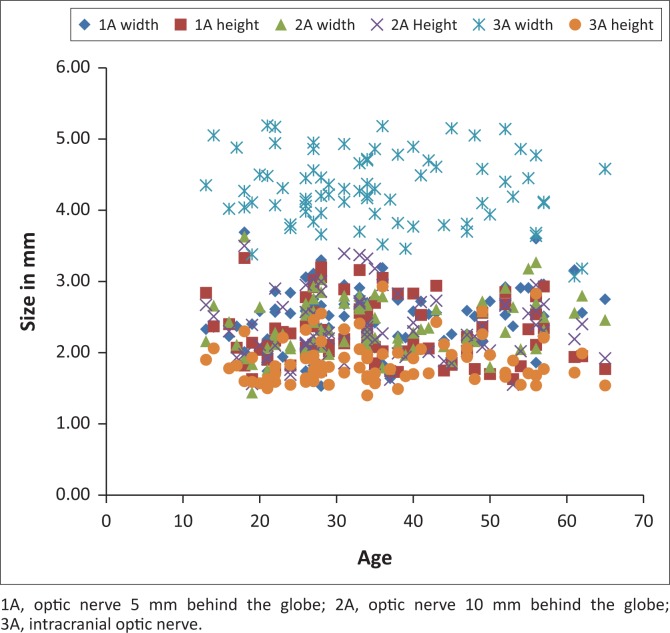
Overall age distribution of optic nerve measurements. No age correlation was demonstrated. The correlation coefficient varied from −0.14 to 0.19.

**FIGURE 5:**
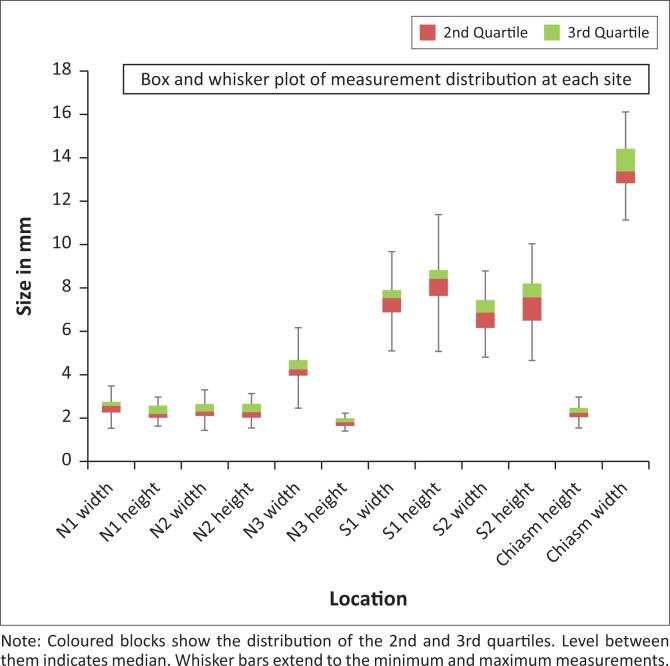
Box and whisker plot demonstrates the overall measurement distribution at different sites.

Measurements of the anterior visual pathway structures were performed for 80 patients. The mean age was 35 years (range: 13–65 years), 49 (61.25%) females and 31 (38.75%) males. Table 1 demonstrates a summary of the overall measurements. There were no measurements with significant statistical differences between males and females. No correlation between age and ON measurements was found; the correlation coefficient was poor, varying from −0.14 to 0.19. A perfect direct correlation would be 1. Figure 4 shows the overall age distribution of all ON measurements. Figure 5 demonstrates the overall measurement distribution at each site.

**TABLE 1:** Overall measurements of the anterior visual pathway structures.

Variable	Dimensions	Overall mean (mm)	± 2 s.d. (mm)	Range (mm)	Female mean (mm)	Male mean (mm)
Nerve 1	w	2.52	1.67–3.37	1.53–3.69	2.52	2.52
	h	2.29	1.43–3.15	1.63–3.33	2.31	2.26
Nerve 2	w	2.37	1.61–3.14	1.44–3.63	2.41	2.31
	h	2.35	1.45–3.24	1.55–3.50	2.36	2.33
Nerve 3	w	4.27	3.21–5.34	2.46–5.19	4.25	4.30
	h	1.88	1.25–2.50	1.40–2.93	1.89	1.85
Sheath 1	w	7.38	5.70–9.07	5.10–9.52	7.31	7.49
	h	8.27	6.07–10.47	5.07–11.45	8.15	8.45
Sheath 2	w	6.81	4.94–8.67	4.81–10.04	6.75	6.91
	h	7.34	4.97–9.71	4.66–10.10	7.27	7.46
Chiasm	w	13.63	11.2–16.06	11.13–16.92	13.51	13.82
	h	2.26	1.60–2.93	1.54–3.38	2.21	2.35

s.d., standard deviation; w, width; h, height; location 1, 5 mm behind the globe; location 2, 10 mm behind the globe; location 3, intracranial segment of the optic nerve.

**FIGURE 6:**
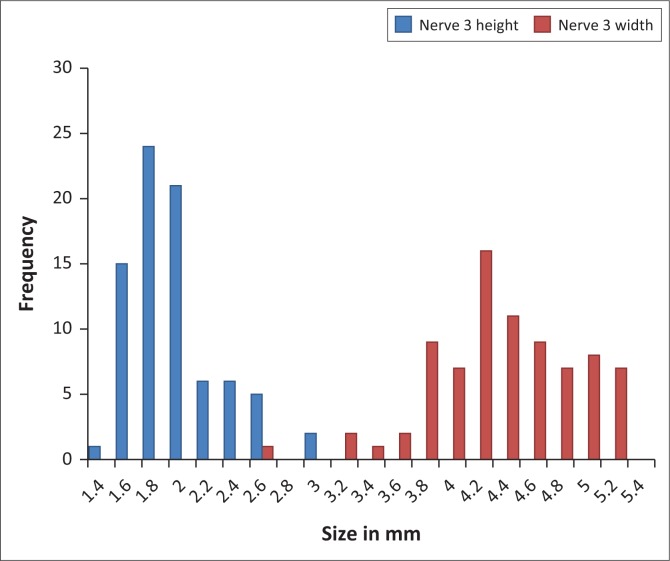
Frequency distribution of the intracranial optic nerve.

**FIGURE 7:**
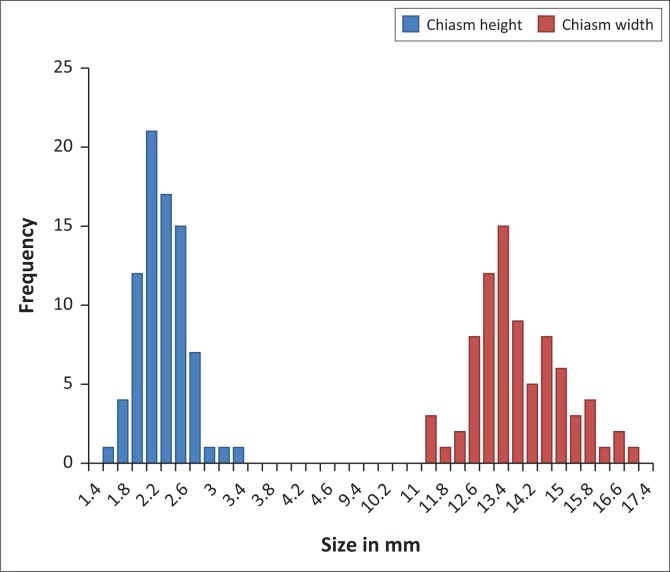
Frequency distribution of the optic chiasm.

Figures 6 and 7 demonstrate the frequency distribution of measurements of the intracranial ON and OC.

**FIGURE 8:**
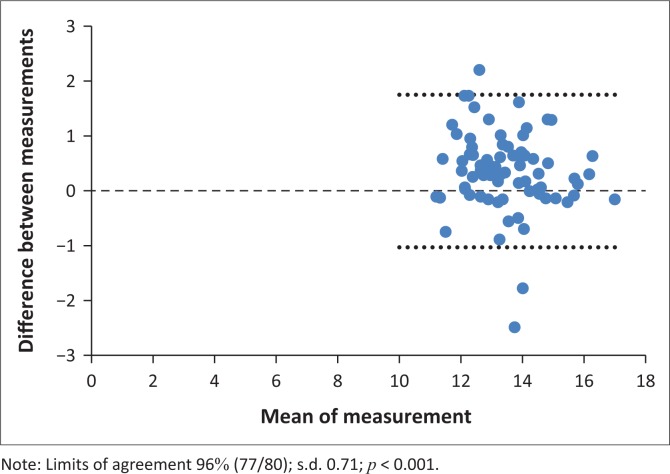
Agreement interval of the optic chiasm width.

**FIGURE 9:**
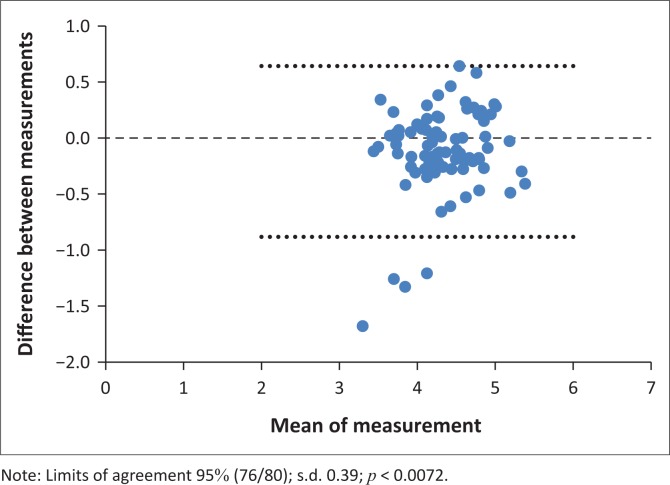
Agreement interval of the intracranial optic nerve width.

Interobserver agreement was tested by the Bland & Altman test (B&A). The best agreement was for the OC width (agreement interval 96%, s.d. 0.71, *p* < 0.001; Figure 8); ON height 5 mm behind the globe (agreement interval 98.8%, s.d. 0.39, *p* < 0.001) and intracranial ON width (agreement interval 95%, s.d. 0.39, *p* < 0.0072; Figure 9).

## Discussion

This study reports normal measurements of the anterior visual pathway structures in adult patients using high-resolution MRI. There were no statistically significant differences between males and females for all the acquired measurements of the anterior visual pathway. This finding is consistent with the previous literature and is expected because there are no known structural or physiological differences of the anterior visual pathway structures between sexes. These observations are consistent with findings by Dolman et al. and Benevento et al.^7,16,17,19,20^ Maresky et al. found that ON measurements increase with age from birth to 12 years of age, followed by a plateau from 12 to 18 years.^14^ The findings of our study showed no correlation between all measurements and age of the selected adult population, confirming that the ON attain their final volume by age 12 years and do not change throughout adult years. This correlates with the findings by Maresky et al. for their 12–18 years’ age category results. Al-Haddad et al. reported an increase in ON diameter with age, especially in the first 2 years of life, confirming that age-related ON size change is a paediatric phenomenon.^21^

The ON diameter in the orbital segment shows progressive decrease, from the retrobulbar to the most posterior segment. There is a corresponding inverse relationship of the ONS diameter. Karim et al. supported this theory in a study by demonstrating significant decrease in the normal ON diameter along its length.^11^ Our results were consistent with this observation. We demonstrated an area difference decrease of 0.16 mm^2^ between 5 mm and 10 mm of the intraorbital ON. This difference is attributed to reduction in the amount of collagen as opposed to neuronal tissue. Karim et al. demonstrated a consistent cross-sectional area of the nervous tissue in the orbital ON, but decreasing amounts of collagen posteriorly on colour densitometry. Interestingly, our results demonstrated a contrasting increase of the intracranial ON area by a factor of 1.56. This may be related to decussation. Prechiasmatic axons have been demonstrated to be ordered in distinct fascicular bundles, different from that observed along the rest of the nerve.^22^ The OC area was substantially larger (up to four times) than the intracranial ON because of decussation.

The anterior visual pathway comprises small structures, including the ON. Obtaining measurements of these structures is often challenging and a tedious exercise because of inherent technical difficulty in measuring small structures. Therefore, identifying reliable points of measurements was an important objective of this study. Parravano et al. suggested that the OC width is the most clinically useful measurement, based on it being the largest and easiest structure to measure. Our study supports this recommendation as the best interobserver agreement was demonstrated for the OC width. Strong agreement was also demonstrated for the intracranial ON width and intraorbital ON height at 5 mm. However, the latter was a difficult measurement to obtain as it requires MPR to view sagittal reformatted images and further straightening techniques to achieve optimal alignment. Therefore, the OC width and intracranial ON width are the most clinically reliable points to obtain measurements along the anterior visual pathway. We recommend that measurements be acquired at these locations. The OC width was the most practical point to perform measurements in our experience. The mean OC width was 13.63 mm (range: 11.13 mm–16.92 mm), intracranial ON width 4.27 mm (range: 2.46 mm–5.19 mm) and intraorbital ON height at 5 mm behind the globe 2.29 mm (range: 1.63 – 3.33 mm). The mean OC width diameter in our study was consistent with that previously reported by Wagner et al. of 14.0 mm, but differed slightly from that reported by Parravano et al. of 15 mm.^5,15^ Recently, Bilal et al. showed comparable results with an OC width of 13.04 mm ±1.21 mm using standard MRI sequences in a coronal plane.^23^

Sonography is regaining popularity in the form of high-resolution transbulbar scanning in the assessment of retrobulbar ONSC, especially in patients requiring serial monitoring of ICP.^24,25,26,27^ Multiple recent studies have assessed its use for measurements of the ONS diameter in the setting of raised ICP. More studies are required to evaluate correlation between ultrasound and MRI for measurements of the ON alone.^24^ Ultrasound offers advantages of ease of availability and repeatability. It also shows good reproducibility, measurement accuracy and observer agreement when measurements are performed 3 mm behind the globe.^27^ The drawbacks of ultrasound include limited penetration to the posterior aspect of the orbit and intracranially. Limited operator skill is also postulated to be a disadvantage as orbital sonography is no longer commonly performed. In view of our study findings showing that the intracranial ON and OC widths are the most clinically reliable points of measurements, MRI is anticipated to remain the imaging modality of choice for most clinical applications.

Traditionally, measurements are obtained manually by using electronic calipers. With the advent of artificial intelligence (AI) and its growing clinical applications, newer techniques of obtaining anterior visual pathway structures are being investigated with promising outcomes. Robert et al. employed a new multi-atlas segmentation method that automatically sections the ON to obtain ON and ONS radii.^28^ Harrigan et al. also recently evaluated a fully automated 3D consistent technique based on a high-resolution, heavily T2-weighted, isotropic MRI technique to obtain ON and ONS radii.^29^ This technique is more anatomically representative because of its 3D nature.

High field strength magnets in a form of 3T are increasingly being used in routine clinical imaging. The 3T magnet strength has clear advantages over the 1.5T, offering superior spatial resolution in depicting the orbital and intracranial anatomy and pathologic findings.^30^ The 7T is still limited to clinical research settings. Use of 3T scanners in similar future studies may yield more positive results of the small orbital ONSC structures as it offers better spatial resolution. In addition, newer mechanical designs may also offer more AI compatibilities, which would support seamless development of newer automated measurement techniques.

## Conclusion

Normal measurements of the anterior visual pathway structures on MRI are best reflected in the larger structures. These are the OC width (mean 13.63 mm, range: 11.13 mm–16.92 mm) and intracranial ON width (mean 4.27 mm, range: 2.46 mm–5.19 mm). Interobserver variability was poor for the orbital ON, ONS, intracranial ON height and OC height. Therefore, we recommend that measurements be obtained for the OC width and intracranial ON width.

## Study limitations

The study participants were recruited at the radiology department. Screening questions were used prior to scanning to exclude abnormal vision and potential central visual pathway pathology. No formal eye examinations were performed. As a result, some participants may have had undetected subclinical visual disturbances.

This was a single institution study with a limited number of potential study recruits, which prolonged the duration of the study by several months, more than initially anticipated.

No specific parameters were set for the electronic functions like zoom, pan and windowing when obtaining the measurements on PACS. This was left to the discretion of each observer to optimise images as they would during routine reporting. Lack of standardisation in this regard may introduce undesirable technical differences between observers, which may create inconsistent viewing conditions affecting the ability to measure small structures with accuracy.

## Recommendations for future research

Further larger population studies are recommended to reliably determine the normal measurements of the smaller anterior visual pathway structures that showed poor interobserver reliability in our study; including the orbital ON, ONS, intracranial OC height and OC height.

## References

[1] Rizzo JF III, editor Neuroimaging of the optic nerve. NANOS 34th Annual Meeting; 2008 Mar 09–13; FL.

[2] Rose GE, Verity DH Neuro-ophthalmology of orbital disease. Handb Clin Neurol. 2011;102:467–491. 10.1016/B978-0-444-52903-9.00023-6 21601077

[3] World Health Organization Blindness and vision impairment [homepage on the Internet]. c2018. Available from: https://www.who.int/news-room/fact-sheets/detail/blindness-and-visual-impairment.

[4] Daniels D, Herfkins R, Gager W, et al Magnetic resonance imaging of the optic nerves and chiasm. Radiology. 1984;152(1):79–83. 10.1148/radiology.152.1.6729139 6729139

[5] Parravano JG, Toledo A, Kucharczyk W Dimensions of the optic nerves, chiasm, and tracts: MR quantitative comparison between patients with optic atrophy and normals. J Comp Assist Tomogr. 1993;17(5):688–690. 10.1097/00004728-199309000-00003 8370820

[6] Votruba M, Leary S, Losseff N, et al MRI of the intraorbital optic nerve in patients with autosomal dominant optic atrophy. Neuroradiology. 2000;42(3):180–183. 10.1007/s002340050041 10772138

[7] Benevento J, Garcia J, Baxter A, Garcia P, Holliday R, Rosen R Optic nerve measurements in normal human eyes by MRI. Invest Ophthalmol Vis Sci. 2004;45(13):2398.

[8] Gass A, Barker G, MacManus D, et al High resolution magnetic resonance imaging of the anterior visual pathway in patients with optic neuropathies using fast spin echo and phased array local coils. J Neurol Neurosurg Psychiatry. 1995;58(5):562–569. 10.1136/jnnp.58.5.562 7745403 PMC1073486

[9] Geeraerts T, Newcombe VF, Coles JP, et al Use of T2-weighted magnetic resonance imaging of the optic nerve sheath to detect raised intracranial pressure. Crit Care. 2008;12(5):R114 10.1186/cc7006 18786243 PMC2592740

[10] Hatase T, Takagi M, Okamoto K, et al Evaluation of the optic nerve complex in the orbit using coronal fast magnetic resonance imaging. Neuro Ophthalmol. 2010;34(2):88–95. 10.3109/01658101003587825

[11] Karim S, Clark RA, Poukens V, Demer JL Demonstration of systematic variation in human intraorbital optic nerve size by quantitative magnetic resonance imaging and histology. Investig Ophthalmol Vis Sci. 2004;45(4):1047–1051. 10.1167/iovs.03-1246 15037567

[12] Lagreze WA, Gaggl M, Weigel M, et al Retrobulbar optic nerve diameter measured by high-speed magnetic resonance imaging as a biomarker for axonal loss in glaucomatous optic atrophy. Investig Ophthalmol Vis Sci. 2009;50(9):4223–4228. 10.1167/iovs.08-2683 19407026

[13] Lenhart PD, Desai NK, Bruce BB, Hutchinson AK, Lambert SR The role of magnetic resonance imaging in diagnosing optic nerve hypoplasia. Am J Ophthalmol. 2014;158(6):1164–1171.e2. 10.1016/j.ajo.2014.08.013 25128595 PMC4252492

[14] Maresky HS, Ben Ely A, Bartischovsky T, et al MRI measurements of the normal pediatric optic nerve pathway. J Clin Neurosci. 2018;48:209–213. 10.1016/j.jocn.2017.11.015 29198418

[15] Wagner AL, Murtagh FR, Hazlett KS, Arrington JA Measurement of the normal optic chiasm on coronal MR images. AJNR Am J Neuroradiol. 1997;18(4):723–726. 9127037 PMC8338490

[16] Weigel M, Lagreze WA, Lazzaro A, Hennig J, Bley TA Fast and quantitative high-resolution magnetic resonance imaging of the optic nerve at 3.0 Tesla. Investig Radiol. 2006;41(2):83–86. 10.1097/01.rli.0000195820.98062.c5 16428977

[17] Kakaria AK Imaging in neuro-ophthalmology: An overview. Oman J Ophthalmol. 2009;2(2):57–61. 10.4103/0974-620X.53033 20671830 PMC2905180

[18] Mashima Y, Oshitari K, Imamura Y, Momoshima S, Shiga H, Oguchi Y High-resolution magnetic resonance imaging of the intraorbital optic nerve and subarachnoid space in patients with papilledema and optic atrophy. Arch Ophthalmol. 1996;114(10):1197–1203. 10.1001/archopht.1996.01100140397006 8859078

[19] Dolman CL, McCormick AQ, Drance SM Aging of the optic nerve. Arch Ophthalmol. 1980;98(11):2053–2058. 10.1001/archopht.1980.01020040905024 7436843

[20] Johnson BM, Miao M, Sadun AA Age-related decline of human optic nerve axon populations. Age. 1987;10(1):5–9. 10.1007/BF02431765

[21] Al-Haddad CE, Sebaaly MG, Tutunji RN, et al Optic nerve measurement on MRI in the pediatric population: Normative values and correlations. Am J Neuroradiol. 2018;39(2):369–374. 10.3174/ajnr.A5456 29217740 PMC7410600

[22] Neveu MM, Holder GE, Ragge NK, Sloper JJ, Collin JRO, Jeffery G Early midline interactions are important in mouse optic chiasm formation but are not critical in man: A significant distinction between man and mouse. Eur J Neurosci. 2006;23(11):3034–3042. 10.1111/j.1460-9568.2006.04827.x 16819992

[23] Bilal DMA Measurements of optic chiasm dimensions using magnetic resonance imaging. Sudan University of Science and Technology; Khartoun, 2018.

[24] Steinborn M, Fiegler J, Kraus V, et al High resolution ultrasound and magnetic resonance imaging of the optic nerve and the optic nerve sheath: Anatomic correlation and clinical importance. Ultraschall Med. 2011;32(6):608–613. 10.1055/s-0029-1245822 21058238

[25] Kalantari H, Jaiswal R, Bruck I, et al Correlation of optic nerve sheath diameter measurements by computed tomography and magnetic resonance imaging. Am J Emerg Med. 2013;31(11):1595–1597. 10.1016/j.ajem.2013.07.028 24054852

[26] Shirodkar CG, Munta K, Rao SM, Mahesh MU Correlation of measurement of optic nerve sheath diameter using ultrasound with magnetic resonance imaging. Indian J Crit Care Med. 2015;19(8):466–470. 10.4103/0972-5229.162465 26321806 PMC4548416

[27] Bäuerle J, Schuchardt F, Schroeder L, Egger K, Weigel M, Harloff A Reproducibility and accuracy of optic nerve sheath diameter assessment using ultrasound compared to magnetic resonance imaging. BMC Neurol. 2013;13(1):187 10.1186/1471-2377-13-187 24289136 PMC4219451

[28] Harrigan RL, Plassard AJ, Mawn LA, Galloway RL, Smith SA, Landman BA Constructing a statistical atlas of the radii of the optic nerve and cerebrospinal fluid sheath in young healthy adults SPIE Medical Imaging, 2017, Orlando, Florida 10.1117/12.2081887 PMC4405797 25914505

[29] Harrigan RL, Smith AK, Mawn LA, Smith SA, Landman BA, editors Improved automatic optic nerve radius estimation from high resolution MRI SPIE Medical Imaging, 2017, Orlando, Florida 10.1117/12.2254370 PMC5521273 28736469

[30] Mafee MF, Rapoport M, Karimi A, Ansari SA, Shah J Orbital and ocular imaging using 3- and 1.5-T MR imaging systems. Neuroimaging Clin N Am. 2005;15(1):1–21. 10.1016/j.nic.2005.02.010 15927858

